# *SULF2* Methylation Is Associated with *In Vitro* Cisplatin Sensitivity and Clinical Efficacy for Gastric Cancer Patients Treated with a Modified FOLFOX Regimen

**DOI:** 10.1371/journal.pone.0075564

**Published:** 2013-10-04

**Authors:** Jie Shen, Jia Wei, Hao Wang, Yang Yang, Guofeng Yue, Lin Wang, Lixia Yu, Li Xie, Xia Sun, Xinyu Bian, Zhengyun Zou, Xiaoping Qian, Wenxian Guan, Baorui Liu

**Affiliations:** 1 The Comprehensive Cancer Centre, Drum Tower Hospital, Medical School of Nanjing University, Clinical Cancer Institute of Nanjing University, Nanjing, Jiangsu, China; 2 Department of General Surgery, Drum Tower Hospital, Medical School of Nanjing University, Nanjing, Jiangsu, China; 3 Nanjing Medical University, Nanjing, Jiangsu, China; Nanjing Medical University, China

## Abstract

**Objective:**

Biomarkers capable of discriminating the patients who are likely to respond to certain chemotherapeutic agents could improve the clinical efficiency. The sulfatases(SULFs) play a critical role in the pathogenesis of a variety of human cancers. Here, we focused our investigation on the prognostic and predictive impact of *SULF2* methylation in gastric cancer.

**Methods:**

Promoter CpG island methylation of *SULF2* was analyzed in 100 gastric cancer samples. The *in vitro* sensitivity to cisplatin, docetaxel, gemcitabine, irinotecan and pemetrexed were determined by histoculture drug response assay(HDRA). Additionally, 56 gastric cancer patients treated with a modified FOLFOX regimen(biweekly oxaliplatin plus 5-FU and folinic acid) were retrospectively analyzed to further evaluate the prognostic and predictive impact of *SULF2* methylation in gastric cancer.

**Results:**

Methylated *SULF2*(*SULF2M*) was detected in 28 patients, while the remaining 72 patients showed unmethylated *SULF2*(*SULF2U*, methylation rate: 28%). Samples with *SULF2U* were more sensitive to cisplatin than those with *SULF2M*(inhibition rate: 48.80% vs. 38.15%, *P* = 0.02), while samples with *SULF2M* were more sensitive to irinotecan than *SULF2U*(inhibition rate: 53.61% vs. 40.92%, *P* = 0.01). There were no association between *SULF2* methylation and *in vitro* sensitivity to docetaxel, gemcitabine and pemetrexed. *SULF2* methylation was found to have a significant association with cisplatin efficacy(*SULF2M*: 57.14%, *SULF2U*: 80.56%, *P* = 0.02) and irinotecan efficacy(*SULF2M*: 89.29%, *SULF2U*: 62.50%, *P* = 0.01). Among the 56 patients receiving the modified FOLFOX regimen, a significant association was observed between survival and *SULF2* methylation status(*SULF2M:* 309 days, 95% CI = 236 to 382 days; *SULF2U:* 481 days, 95% CI = 418 to 490 days; *P* = 0.02). Multivariate analysis revealed that *SULF2* methylation was an independent prognostic factor of overall survival in gastric cancer patients treated with platinum-based chemotherapy.

**Conclusion:**

*SULF2* methylation is negatively associated with cisplatin sensitivity *in vitro*. *SULF2* methylation may be a novel prognostic biomarker for gastric cancer patients treated with platinum-based chemotherapy.

## Introduction

Gastric cancer is one of the most frequent causes of cancer-related death worldwide [1], [2]. Multimodal treatment protocols, mainly based on platinum and 5-fluorouracil (5-FU), have been shown to prolong patient survival; however, with any combination of chemotherapeutic agents, the response rate is only approximately 30%–50% [3], [4]. In an attempt to improve the clinical efficiency, it is important and necessary to identify biomarkers capable of discriminating the patients who are likely to respond to certain chemotherapeutic agents [4]–[9].

Heparan sulfate proteoglycans (HSPGs) are coreceptors for heparin-binding growth factors and cytokines distributed on the cell surface and in the extracellular matrix. Two isoforms of the extracellular heparan sulfate 6-O-endosulfatases (SULFs), SULF1 and SULF2, have been discovered in mammals. Both proteins are secreted to the cell surface to modulate the sulfation of HSPGs [10]. Although previous reports have unequivocally highlighted the critical role that SULFs play in the pathogenesis of a variety of human cancers, the opinions on the role of SULFs in cancer development have been somewhat polarized. Several evidences show that SULFs are negative regulators of tumor cell growth, and that overexpression of SULFs in tumor cells inhibits cell growth by deregulating several factors, including FGF-2, HB-EGF and HGF [11]–[13]. Other studies hold the view that SULFs are positive regulators of oncogenetic signaling pathways, including Wnt, BMP, Hedgehog and GDNF [14]–[16], and increased expression of SULFs is prevalent in various cancers, including gastric, hepatocellular, pancreatic and breast cancers, non-small cell lung cancer (NSCLC) and head and neck tumors [10], [13], [17]–[20]. High expression of SULF2 has been linked to poor survival in patients with hepatocellular carcinoma and NSCLC [18], [20]. The available evidence on the methylation status and expression levels of *SULFs* in gastric cancer, however, are non-conclusive, and the prognostic or predictive value of *SULFs* for chemosensitivity prediction remains unknown.

In this study, we have analyzed the promoter CpG island methylation of *SULF2*, the gene encoding the SULF2 endosulfatase, and its association with *in vitro* sensitivity to cisplatin, docetaxel, gemcitabine, irinotecan, and pemetrexed in 100 human gastric cancer samples. To this end, we performed a series of *in vitro* sensitivity tests on freshly-removed gastric tumor tissues and evaluated the possible use of *SULF2* methylation status for predicting the chemotherapeutic efficacy of there agents. Then, we retrospectively analyzed the *SULF2* methylation in a cohort of 56 gastric cancer patients treated with a modified FOLFOX regimen and concluded that *SULF2U* serves as an independent prognostic biomarker in gastric cancer patients treated with a modified FOLFOX regimen.

## Materials and Methods

### Ethics Statement

All research involving human participants have been approved by the Human Research Protective Committee of Drum Tower Hospital Affiliated to Medical School of Nanjing University and written informed consent was obtained from all patients.

### Patients and Tissue Samples

Enrolled patients were those undergoing the gastrectomy in the Department of General Surgery of the Drum Tower Hospital, Nanjing, China during the period from August 2010 to October 2011. Eligibility criteria for enrollment into the study included the following parameters: (1) age >18 years; (2) histologically confirmed gastric adenocarcinoma, mucinous or signet ring cell adenocarcinoma; (3) no previous or concomitant malignancies other than gastric cancer; (4) no previous history of chemotherapy or radiotherapy, either adjuvant or palliative; and (5) adequate function of all major organs. Tissue samples were extracted from 100 freshly-removed gastric tumors. Each tumor tissue was divided into two parts: (1) one part was kept in 4°C Hanks’ balanced salt solution with 1% penicillin/streptomycin after collection, and then sent to the laboratory within 15 min in 4°C, for *in vitro* evaluation of chemosensitivity by histoculture drug response assay (HDRA) [21], [22]; (2) the remaining part was made into formalin-fixed paraffin-embedded (FFPE) tumor blocks for pathological observation and methylation detection. We retrospectively reviewed the patients’ medical records and surgery reports to identify clinical and histopathological data, including sex, histology, tumor site, stage, histological grade and lymph node metastasis. Tumors were classified according to the International Union Against Cancer (UICC) TNM Classification. Written informed consent was obtained from all patients and the protocols for this study were approved by the Human Research Protective Committee of the Drum Tower Hospital.

### HDRA

HDRA procedures were performed as previously reported by Furukawa and colleagues [21], [22]. Briefly, the fresh tumor tissues were washed twice with Hanks’ balanced salt solution and minced into small pieces of approximately 10 mg in weight and 0.5 mm in diameter, which were then placed on prepared collagen (Health Design, Rochester, NY) surfaces in 24-well microplates. Optimal concentrations of the drugs used to distinguish *in vitro* sensitivity and resistance were 20 ug/ml for cisplatin [23], 100 ug/ml for docetaxel [22], 50 ug/ml for gemcitabine [23], 20 ug/ml for irinotecan [23], and 400 ug/ml for pemetrexed [5], according to its peak plasma concentration in patients. Cisplatin, docetaxel, and irinotecan were obtained from Jiangsu Hengrui Medicine Company (Jiangsu, China), while pemetrexed and gemcitabine were obtained from Eli Lilly and Company (Shanghai, China). 8 parallel culture wells were used for each drug concentration, as well as for control. Plates were incubated for 7 days at 37°C in the presence of drugs dissolved in RPMI 1640 medium containing 20% fetal calf serum and left in a humidified atmosphere containing 95% air–5% CO_2_. After histoculture, 100 µl of type I collagenase (0.1 mg/ml, Sigma, Shanghai, China) and 100 µl of 3-(4,5-Dimethyl-2-thiazotyl)-2,5-diphenyl-2H- tetrazolium bromide (MTT) solution (5 mg/ml, Sigma, Shanghai, China) were added to each culture well and incubated for another 16 hours. After extraction with dimethyl sulfoxide, the absorbance of the solution in each well was read at 540 nm.

### Evaluation of Sensitivity to Anti-cancer Agents in HDRA

The absorbance per gram of cultured tumor tissue was calculated from the mean absorbance of tissue from 8 parallel culture wells, and the tumor tissue weight was determined before culture. The inhibition rate of each anti-cancer agent was calculated by using the following formula: Inhibition rate (%) = (1–T/C) ×100, where T is the mean absorbance of treated tumor / Weight and C is the mean absorbance of control tumor / Weight. The cut-off inhibition rates used to distinguish the sensitive and the resistant cases were adopted at 30%, 40%, 50%, and 60% for each drug tested. The *in vitro* efficacy rate was also estimated based on the cut-off inhibition rate as follows: efficacy rate (%) = number of sensitive cases/total number of cases.

### Patients’ Chemotherapy

Of all patients, 56 with Eastern Cooperative Oncology Group (ECOG) performance status (PS) ≤2 received a modified FOLFOX (a combination of 5-FU and platinum) chemotherapy regimen after resection of primary tumors as follows: oxaliplatin 85 mgm^−2^ plus folinic acid 200 mgm^−2^ as a 2 h infusion on day 1, followed by a 22 h infusion of 5-FU 2000 mgm^−2^ on days 1 and 2, every two weeks. These 56 patients were further followed up and their survival time was calculated from the date of diagnosis to the date of the last follow-up or death from any cause.

### DNA Extraction and Modification

Three 7-µm sections were prepared from primary tumor blocks that contained at least 80% tumor cells. After hematoxylin-eosin staining, the cancerous parts were microdissected and transferred into a microcentrifuge tube. DNA was isolated routinely and then was chemically modified by sodium bisulphite to convert all unmethylated cytosines to uracils while leaving methylcytosines unaltered [18]. Then they were stored at −20°C for further analysis.

### Methylation-Specific Polymerase Chain Reaction (MSP)

MSP was performed to determine the methylation status of *SULF2* using the ABI Prism 7300HT Sequence Detection System (Applied Biosystems). Each PCR reaction contained genomic DNA 2 µl, SYBR Green PCR Mix (TaKaRa, Japan) 10 µl, water 7.7 µl, and primers 0.15 µl (10 µmol/l). The PCR conditions were 95°C for 10 min, followed by 45 cycles at 59°C for 30 s, 72°C for 30 s and 95°C for 30 s. Primers for *SULF2* methylated PCR (TaKaRa, Japan) were as follows: forward 5′ TAAGTGTTTTTTTTATAGCGGC 3′, reverse 5′TACCGTAATTTCCGCTATC 3′. Primers for *SULF2* unmethylated PCR (TaKaRa, Japan) were as follows: forward 5′ GTTTATAAGTGTTTTTTTATAGTGGT3’, reverse 5′TACCATAATTTCCACTATCCCT 3′. Each batch of reaction included a positive control from Methyltransferase (M.SssI)-treated human genomic DNA (fully methylated), a negative control from DNA samples which had been confirmed as unmethylated and another negative control without DNA. All tests were performed in duplicate. Results were validated for selected samples through combined bisulfite modification and bisulfite sequencing.

### Statistical Analysis

Values were expressed as means ± standard deviation. Differences in inhibition rates between groups were evaluated using the t-test. The possible associations of SULF2 methylation with clinical characteristics and *in vitro* chemosensitivity efficacy were analyzed using the Fisher’s exact probability test. All statistical tests were two-sided, and a P value of less than 0.05 was considered as statistical significance. Statistical analysis was performed using the SPSS, version 16.0.

## Results

### Patients’ Characteristics

The characteristics of all patients are shown in Table 1. The majority of patients were males (73%), and the predominant histology was adenocarcinoma (79%). In 35 (35%) patients, the tumor was located in the distal stomach, in 38 (38%) in the proximal stomach, and in 27 (27%) in the whole stomach. Sixty-three (63%) patients had stage III or IV disease. Lymph node metastases were present in 75 (75%) patients. Methylated *SULF2* (*SULF2M*) was detected in 28 patients, while the remaining 72 patients showed unmethylated *SULF2* (*SULF2U*, methylation rate: 28%). The RT-PCR amplification curves of *SULF2M* and *SULF2U* were shown in Figure S1. There was no association between *SULF2* methylation and any of the patients’ characteristics, including sex, histology, tumor site, stage, histological grade and lymph node metastasis.

**Table 1:** Patient characteristics.

Characteristic	Patients	*SULF2*		
	N = 100, N (%)	*SULF2M*	*SULF2U*	*P* Value
Age				
>62	52 (52%)	14	38	0.83
≤62	48 (48%)	14	34	
Sex				
Male	73 (73%)	21	52	0.49
Female	27 (27%)	7	20	
Histology				
Adenocarcinoma	79 (79%)	24	55	0.59
Mucinous	11 (11%)	2	9	
Signet ring cell	10 (10%)	2	8	
Tumor Site				
Distal	35 (35%)	6	29	0.21
Proximal	38 (38%)	13	25	
Whole stomach	27 (27%)	9	18	
Stage				
I,II	37 (37%)	8	29	0.36
III, IV	63 (63%)	20	43	
Histological grade				
2	27 (27%)	5	22	0.23
3	44 (44%)	16	28	
Mixed 2–3	29 (29%)	7	22	
Lymph node metastasis				
No	25 (25%)	5	20	0.44
Yes	75 (75%)	23	52	

### *In vitro* Efficacy Rate of Tested Drugs

**Figure 1:**
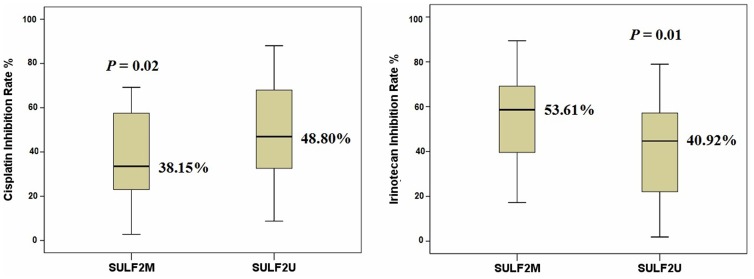
Samples with *SULF2U* were more sensitive to cisplatin and those with *SULF2M* (48.80% vs. 38.15%, *P* = 0.02, n = 100, t-test); samples with *SULF2M* were more sensitive to irinotecan than *SULF2U* (53.61% vs. 40.92%, *P* = 0.01, n = 100, t-test). The median is the central line in each box.

*In vitro* sensitivity to cisplatin, docetaxel, gemcitabine, irinotecan and pemetrexed was successfully tested in all the samples. The mean inhibition rates for each tested drug are listed in Table 2. Samples with *SULF2U* were more sensitive to cisplatin than those with *SULF2M* (48.80% vs. 38.15%, *P* = 0.02), while samples with *SULF2M* were more sensitive to irinotecan than *SULF2U* (53.61% vs. 40.92%, *P* = 0.01, Table 2 and Figure 1). There was no association between *SULF2* methylation and *in vitro* sensitivity to docetaxel, gemcitabine or pemetrexed (Table 2). As shown in Table 3, there was no association between the inhibition rates observed for the anti-cancer agents and any of the clinical characteristics.

**Table 2:** Descriptive statistics of investigated drugs.

Chemotheraputic agents	Number of samples	Inhibition rate (%)		*SULF2*		
		Mean ± SD	Range	*SULF2M* (n = 28)	*SULF2U* (n = 72)	*P* Value
Cisplatin	100	45.94±21.02	2.76–88.02	38.15% (30.27%–46.03%)	48.80% (43.94%–53.65%)	0.02
Docetaxel	100	45.69±22.96	4.19–88.40	45.25% (35.89%–54.60%)	45.86% (40.41%–51.32%)	0.91
Gemcitabine	100	44.72±21.78	3.55–88.97	48.13% (38.85%–57.41%)	43.45% (37.88%–49.02%)	0.38
Irinotecan	100	46.22±22.59	1.86–89.42	53.61% (45.17%–62.04%)	40.92% (35.74%–46.10%)	0.01
Pemetrexed	100	52.79±22.40	3.60–89.46	54.78% (45.18%–64.39%)	52.01% (46.95%–57.07%)	0.58

**Table 3:** Association between inhibition rates of each anti-cancer agent and clinical characteristics.

Characteristic	PatientsN = 100 N (%)	Cisplatininhibition rate %	Doctaxelinhibition rate %	Gemcitabine inhibition rate %	Irinotecanl inhibition rate %	Pemtrexed inhibition rate %
Age						
>62	52 (52%)	45.20±20.78	44.79±21.64	46.31±22.23	42.60±23.08	51.21±22.77
≤62	48 (48%)	46.48±21.48	46.70±24.55	43.01±21.44	46.50±22.12	54.50±22.10
Sex						
Male	73 (73%)	46.71±21.89	47.81±21.99	47.33±20.74	44.41±21.39	52.65±22.23
Female	27 (27%)	43.39±18.64	39.90±24.98	38.06±23.38	44.64±26.01	53.16±23.27
Histology						
Adenocarcinoma	79 (79%)	48.71±19.80	44.48±22.33	45.89±21.53	42.66±21.67	54.11±23.02
Mucinous	11 (11%)	28.86±17.71	36.06±24.58	41.05±22.72	43.14±21.90	53.22±20.15
Signet ring cell	10 (10%)	50.46±12.87	47.32±25.05	39.63±24.24	37.55±31.13	55.62±22.95
Tumor Site						
Distal	35 (35%)	52.20±19.38	47.84±22.92	41.67±20.12	42.28±24.22	49.22±23.55
Proximal	38 (38%)	41.86±19.31	39.52±21.90	48.04±23.13	42.60±23.11	57.61±19.91
Whole stomach	27 (27%)	46.81±20.07	45.04±23.65	43.87±22.19	41.35±20.35	55.37±24.51
Stage						
I,II	37 (37%)	46.94±21.58	45.54±24.10	38.77±20.03	42.67±22.45	53.86±23.37
III, IV	63 (63%)	46.71±18.94	42.98±22.11	48.12±22.19	41.87±22.85	54.36±22.18
Histological grade						
2	27 (27%)	46.83±22.49	45.73±22.27	50.07±20.02	43.44±22.71	55.17±19.31
3	44 (44%)	42.48±18.35	38.12±22.61	39.72±22.06	40.69±22.89	56.11±20.83
Mixed 2–3	29 (29%)	53.40±18.03	50.93±22.01	47.06±22.25	43.17±22.80	50.20±27.77
Lymph node metastasis						
No	25 (25%)	48.82±23.19	48.81±24.53	39.01±22.98	42.80±22.84	52.53±20.81
Yes	75 (75%)	44.81±20.31	44.67±22.51	46.47±21.28	45.03±22.63	52.87±23.04

Data are expressed as mean ± standard deviation.

**Figure 2:**
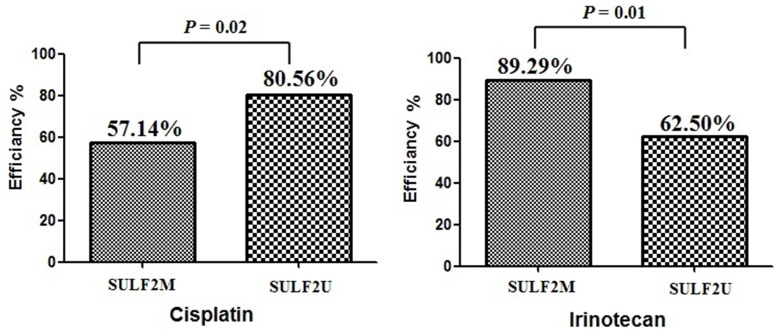
Using the 30% inhibition rate as cut-off, *SULF2* methylation was found to have a significant association with cisplatin efficacy (*SULF2M*: 57.14%, *SULF2U*: 80.56%, *P* = 0.02) and irinotecan efficacy (*SULF2M*: 89.29%, *SULF2U*: 62.50%, *P* = 0.01).

To further investigate the possible relationship between *SULF2* methylation and cisplatin or irinotecan sensitivity, the *in vitro* efficacy rate of each drug concentration was examined setting up different cut-off inhibition rates (Table 4). Four different cut-off values were adopted: 30%, 40%, 50% and 60%. At the cut-off of 30% inhibition rate, *SULF2* methylation was found to have a significant association with cisplatin efficacy (*SULF2M*: 57.14%, *SULF2U*: 80.56%, *P* = 0.02) and irinotecan efficacy (*SULF2M*: 89.29%, *SULF2U*: 62.50%, *P* = 0.01, Figure 2 and Table 4). At the cut-off of 40%, 50% and 60% inhibition rate, there was a trend that the *SULF2M* samples showed higher irinotecan efficacy, but lower cisplatin efficacy than the *SULF2U* samples (Table 4).

**Table 4:** Relationship between cut-off inhibition index and *in vitro* efficacy rate by means of HDRA.

Chemotheraputic agents and SULF2 methylation	Cut-off inhibition rate															
	30%				40%				50%				60%			
	Sensitive (sample number)	Resistant (sample number)	Efficacy rate (%)	*P* value	Sensitive (sample number)	Resistant (sample number)	Efficacy rate (%)	*P* value	Sensitive (sample number)	Resistant (sample number)	Efficacy rate (%)	*P* value	Sensitive (sample number)	Resistant (sample number)	Efficacy rate (%)	*P* value
**Cisplatin (20 ug/ml)**																
**SULF2M**	16	12	57.14	0.02	12	16	42.86	0.07	11	17	39.29	0.26	6	22	27.27	0.46
**SULF2U**	58	14	80.56		46	26	63.89		39	33	54.17		22	50	44.00	
**Irinotecan (20 ug/ml)**																
**SULF2M**	25	3	89.29	0.01	21	7	75.00	0.07	16	12	57.14	0.11	12	16	42.86	0.05
**SULF2U**	45	27	62.50		38	34	52.78		27	45	37.50		16	56	22.22	

### Association of *SULF2* Methylation with Clinical Response to Chemotherapy

**Figure 3:**
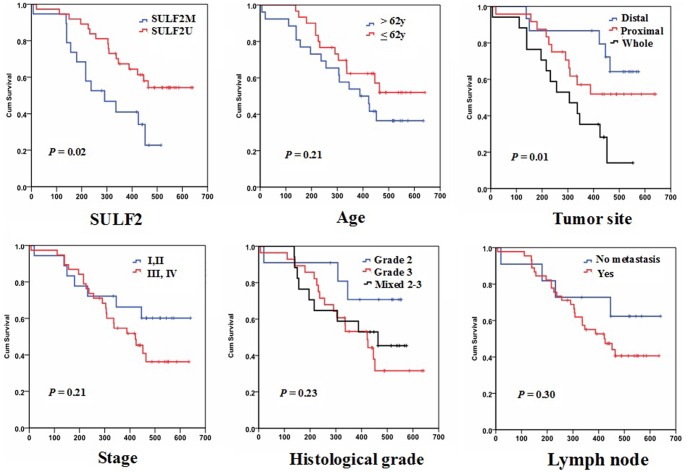
Kaplan–Meier estimates of overall survival by *SULF2* methylation status and clinical characteristics.

Among the 56 patients receiving the modified FOLFOX regimen, the median survival time was 434 days (range: 111–641 days). A significant association was observed between survival and tumor site (*P* = 0.01). No other association between clinical characteristics and survival was found (Table 5 and Figure 3). *SULF2M* was detected in 19 patients, while *SULF2U* was found in 37 patients (*SULF2* methylation rate: 34%). A significant association was observed between survival and *SULF2* methylation status. The median overall survival was 309 days (95% CI = 236 to 382 days) for patients with *SULF2M*, and 481 days (95% CI = 418 to 490 days) for those with *SULF2U* (*P = *0.02, Figure 3). Using both univariate and multivariate Cox proportional hazard analysis that took into account age, sex, tumor site, stage, histological grade, lymph node metastasis and *SULF2* methylation as covariates, tumor site and *SULF2* methylation remained significant markers of overall survival in gastric cancer patients treated with platinum-based chemotherapy (Table 6).

**Table 5:** Clinical factors associated with overall survival.

Characteristic	No. of Patients N = 56	Median survival time (days)	P Log-rank test
**Age, y**			
>62	26	391	0.21
≦62	30	471	
**Sex**			
Male	42	399	0.48
Female	14	469	
**Histology**			
Adenocarcinoma	43	426	0.93
Mucinous	7	470	
Signet ring cell	6	416	
**Tumor Site**			
Distal stomach	15	414	0.01
Proximal stomach	24	366	
Whole stomach	17	229	
**Stage**			
I, II	18	473	0.21
III, IV	38	411	
**Histological grade**			
2	11	459	0.23
3	28	401	
Mixed 2–3	17	395	
**Lymph node metastasis**			
No	11	485	0.30
Yes	45	420	
***SULF2***			
Methylation	19	309	0.02
Unmethylation	37	481	

**Table 6:** Univariate and multivariate Cox proportional hazard analysis of factors associated with overall survival.

Variables	Univariate			Multivariate	
	No.	HR (95% CI)	P	HR (95% CI)	P
**Age, y**					
>62	26	1(ref.)		1(ref.)	
≦62	30	0.632 (0.304–1.314)	0.21	0.509 (0.223–1.165)	0.11
**Sex**					
Male	42	1(ref.)		1(ref.)	
Female	14	0.809 (0.296–1.788)	0.49	0.363 (0.124–1.066)	0.07
**Histology**					
Adenocarcinoma	43	1(ref.)		1(ref.)	
Mucinous	7	0.849 (0.292–2.467)	0.76	0.206 (0.052–0.818)	0.03
Signet ring cell	6	1.116 (0.333–3.737)	0.86	0.568 (0.131–2.460)	0.45
**Tumor Site**					
Distal stomach	15	1(ref.)		1(ref.)	
Proximal stomach	24	1.813 (0.626–5.248)	0.27	3.381 (0.794–14.395)	0.10
Whole stomach	17	3.975 (1.390–11.363)	0.01	8.105 (1.814–36.218)	0.01
**Stage**					
I, II	18	1(ref.)		1(ref.)	
III, IV	38	1.716 (0.729–4.040)	0.22	2.321 (0.487–11.050)	0.29
**Histological grade**					
2	11	1(ref.)		1(ref.)	
3	28	2.810 (0.820–9.631)	0.10	6.354 (1.134–35.601)	0.05
Mixed 2–3	17	2.287 (0.619–8.456)	0.22	4.401 (0.891–21.751)	0.07
**Lymph node metastasis**					
No	11	1(ref.)		1(ref.)	
Yes	45	1.732 (0.601–4.988)	0.31	1.018 (0.216–4.791)	0.98
**SULF2**					
Methylation	19	1(ref.)		1(ref.)	
Unmethylation	37	0.413 (0.197–0.866)	0.02	0.525 (0.215–1.280)	0.04

In this multivariate analysis, age, sex, tumor site, stage, histological grade, lymph node metastasis and *SULF2* methylation were included.

## Discussion

Although previous studies have demonstrated the involvement of SULF2 in cancer pathogenesis, its value for chemosensitivity prediction remains unclear. This study focuses on the promoter CpG island methylation of *SULF2* as a potential biomarker in gastric cancer. The major findings of the present study demonstrate that: (i) the rate of *SULF2M* in human gastric cancer is around 30%; (ii) the first evidence for the *SULF2U* is associated with cisplatin sensitivity in cancer; (iii) evidence for the *SULF2M* is associated with irinotecan sensitivity in gastric cancer; (iv) a retrospective study and validation for *SULF2* methylation in a cohort of 56 patients with gastric cancer, which allowed us to discover that *SULF2U* is an independent prognostic biomarker in gastric cancer patients treated with a modified FOLFOX regimen.

In previous studies of gastric cancer, the findings on methylation status and expression levels of *SULFs* were inconclusive. In one study, only 13 early-stage breast and gastric cancers, most of which were stage I, were analyzed by semi-quantitative RT-PCR for *SULF1* expression [24]. It was shown that low expression of *SULF1* was prevalent in these two types of cancer. In another study, the expression of both *SULF1* and *SULF2* mRNA was determined by real-time RT-PCR for a large cohort of gastric cancer tissues, finding that *SULFs* were expressed at higher levels in gastric cancer as compared with normal tissues. In the current study we found by examining the *SULF2* methylation in 100 gastric cancer samples, that the rate of *SULF2M* was approximately 30%, which indicated that predominate gastric cancer were *SULF2U*. Recent studies by integrated genomic analyses revealed that *SULF2* acts as a downstream effector of p53, and that activation of p53 could lead to the *SULF2U* and up-regulation of *SULF2*. The possible link between p53 and SULF2 in growth factor signaling pathway suggested a possible role for SULF2 in cancer development and cancer patients’ outcome [25]. The relationship between *SULF2* unmethylation and *SULF2* up-regulation needs to be further tested in these samples. The different expression levels and methylation status of *SULF2* between tumor and normal tissue also need to be further verified.

Studies on *SULF2* methylation as chemosensitivity predictor are scarce. Methylation of the *SULF2* promoter has been associated with better survival of resected lung adenocarcinoma patients, and also with a marginal improvement in survival of advanced NSCLC patients receiving standard chemotherapy (hazard ratio = 0.63, *P* = 0.07) [18]. Subsequent studies demonstrated that NSCLC cell lines with *SULF2M* were 134-fold more sensitive to topoisomerase I inhibitor than those with *SULF2U*. In the current study, we have demonstrated that gastric tumors with *SULF2M* are more sensitive to irinotecan than those with *SULF2U*. Furthermore, we demonstrated for the first time that *SULF2* methylation is also a potential predictive biomarker for cisplatin efficacy. Gastric tumors with *SULF2U* were more sensitive to cisplatin than those with *SULF2M*, and gastric cancer patients with *SULF2U* showed lower mortality when receiving platinum-based chemotherapy. Although several predictive biomarkers have been identified for cisplatin, such as ERCC1 [7], BRCA1 [4], [26], [27] and XRCC1 [28]–[30], considering the relatively low response rates of the commonly used platinum/5FU-based neoadjuvant treatment protocol for advanced gastric carcinoma patients, the identification of biomarkers to predict response is urgently needed. The discovery of a novel predictive biomarker that can be examined by methylation analysis is intriguing and supplemental. The reason why *SULF2* unmethylation increases tumor sensitivity to cisplatin may lie on ubiquitin conjugating enzymes (UBE). It has been reported that *SULF2* methylation results in increased expression of UBE [18]. UBE added ubiquitin to certain lysine residues and was involved in DNA repair, mutagenesis and cell proliferation. Overexpression of certain UBEs, like RAD6B, could result in significant resistance to cisplatin [31]. Further studies should be carried out to investigate the molecular mechanism of *SULF2M* induced cisplatin resistance.

A significant synergistic effect of cisplatin and DNA methyltransferase (DNMT) inhibitors 5-aza-dC (DAC) on cell viability was observed in the cisplatin-resistant AGS cell line but not in the cisplatin-sensitive MKN28 cell line [32]. Data from analyzing colony formation capability showed that knockdown of DNMT1 caused an increase in cisplatin sensitivity [32]. The molecular mechanism remains unclear. However, the relationship between *SULF2* methylation and cisplatin sensitivity may partially explain this phenomenon. Treatment of gastric cancer with DNMT inhibitors could result in demethylation of *SULF2* and consequently increase the sensitivity to cisplatin. Thus, in addition to the predictive impact, our data also supports the inclusion of a DNMT inhibitor in current treatment protocols for at least a subset of gastric cancer patients.

In conclusion, our study provides novel evidences that *SULF2* methylation is negatively associated with cisplatin sensitivity *in vitro*. *SULF2* methylation is a potential prognostic biomarker for gastric cancer patients treated with platinum-based chemotherapy.

## Supporting Information

Figure S1 **The RT-PCR amplification curves of SULF2M and SULF2U.** The red curve stands for amplification of SULF2M, and the blue curve stands for amplification of SULF2U. Figure S1a shows the amplification curves of sample with SULF2M; Figure S1b shows the amplification curves of sample with SULF2U.
(TIF)
