# Supplementary figures and images for: SULF2 Methylation Is Associated with In Vitro Cisplatin Sensitivity and Clinical Efficacy for Gastric Cancer Patients Treated with a Modified FOLFOX Regimen
Source: PLoS One. 2013 Oct 4;8(10):e75564. doi: 10.1371/journal.pone.0075564 (PMC3790846; doi:10.1371/journal.pone.0075564)

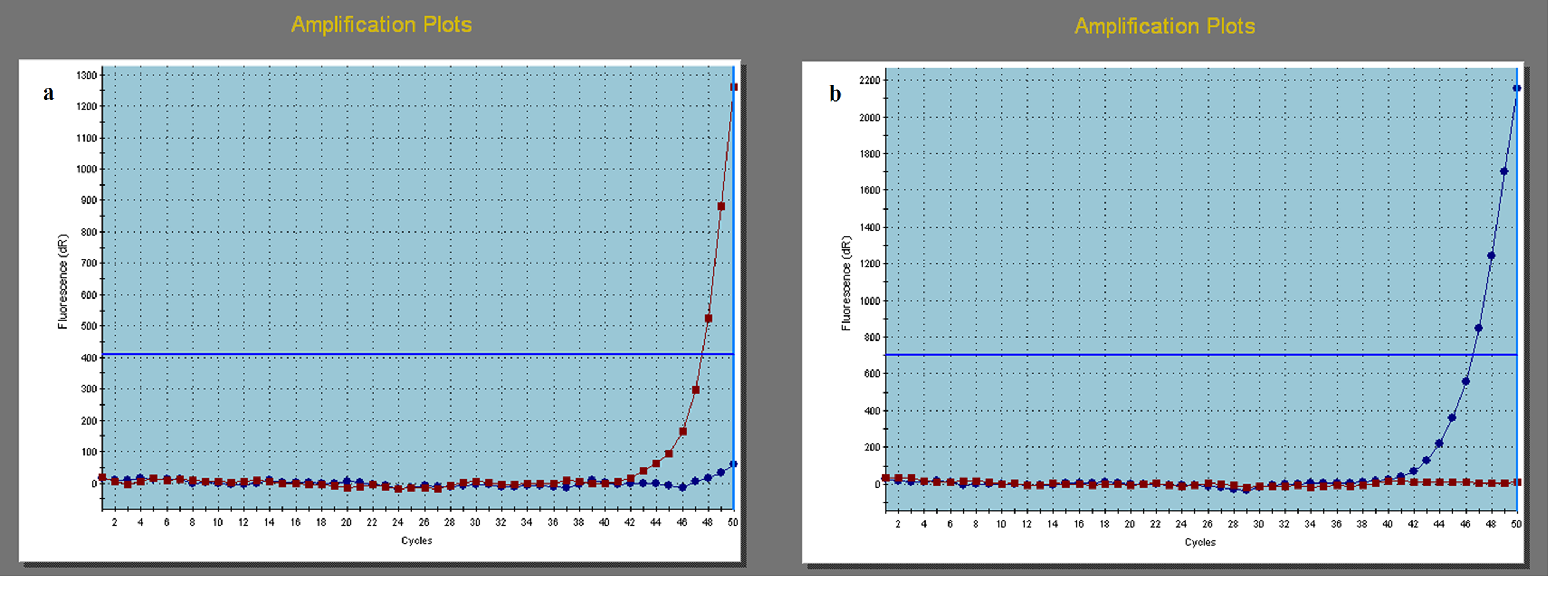

Supplement: Figure S1 — The RT-PCR amplification curves of SULF2M and SULF2U. The red curve stands for amplification of SULF2M, and the blue curve stands for amplification of SULF2U. Figure S1a shows the amplification curves of sample with SULF2M; Figure S1b shows the amplification curves of sample with SULF2U. (TIF) [file pone.0075564.s001.tif]
